# Effective communication of COVID-19 vaccine information to recently-arrived culturally and linguistically diverse communities from the perspective of community engagement and partnership organisations: a qualitative study

**DOI:** 10.1186/s12913-023-09836-3

**Published:** 2023-08-21

**Authors:** Kara Dickson, Craig Aboltins, Janet Pelly, Rebecca Leigh Jessup

**Affiliations:** 1 School of Population and Global Health, The University of Melbourne, Melbourne, VIC Australia; 2 Department of Infectious Diseases, Northern Health, Epping, VIC 3076 Australia; 3 Adjunct Associate Professor, Department of Medical Education, The University of Melbourne, Melbourne, Australia; 4 Northern Health, Epping, VIC 3076 Australia; 5 Director of Research and Evaluation, Staying Well. Northern Health, Epping, VIC 3076 Australia; 6 Adjunct Research Fellow, School of Allied Health, Human Services and Sport, La Tobe University, Bundoora, Australia; 7 Adjunct Research Fellow, School of Rural Health, Monash University, Warragul, Australia

**Keywords:** Vaccine hesitancy, Migrant, Refugee, Asylum seeker, Health communication, Qualitative research

## Abstract

**Background:**

In many high-income countries, COVID-19 has disproportionately impacted Culturally and Linguistically Diverse (CALD) communities. Barriers to engaging with essential health messaging has contributed to difficulties in following public health advice and exacerbated existing inequity in Australia. Research suggests that recently-arrived CALD populations are particularly vulnerable to misinformation and are more likely to experience vaccine hesitancy. The aim of this study was to explore the barriers and enablers to COVID-19 vaccination among recently-arrived CALD communities in Melbourne’s outer north and identify strategies to reduce hesitancy in this population.

**Methods:**

Semi-structured interviews were conducted with representatives from community organisations working with recently-arrived CALD communities in Melbourne’s north. This included a mix of peer (from the community) and health care workers.

**Results:**

Fifteen participants from community organisations participated in interviews. Thematic analysis identified four themes; (1) trusted sources, (2) accurate and culturally sensitive information, (3) supported pathways and (4) enablers to vaccination.

**Conclusions:**

Participants reported a perceived lack of accurate, culturally sensitive health information and service provision as key barriers to vaccination in recently-arrived CALD communities. Participants identified a range of perceived enablers to increasing vaccination uptake in the communities they work with, including utilising established channels of communication and harnessing the communities’ strong sense of collective responsibility. Specific strategies to reduce vaccine hesitancy included identifying and utilising trusted sources (e.g. faith leaders) to disseminate information, tailoring health messages to address cultural differences, providing opportunities to contextualise information, and modifying service delivery to enhance cultural sensitivity. There is an urgent need for increased efforts from health and government agencies to build sustainable, collaborative relationships with CALD communities.

## Background

In many high-income countries, COVID-19 has disproportionately impacted Culturally and Linguistically Diverse (CALD) communities [1, 2]. These communities have suffered higher than average COVID-19 morbidity and mortality, believed to be related to higher rates of poverty, greater household sizes and employment with a high exposure risk [2]. Language barriers, health information seeking from overseas sources, and a lack of culturally appropriate engagement strategies for this population contributed to difficulties in following public health advice and exacerbated existing inequity in Australia [3–5]. Recent research conducted in the north of Melbourne found that those whose preferred language was not English were 11 times more likely to believe misinformation about how to prevent the spread of COVID-19 [6].

Accurate and easily accessible public health advice is essential in combating vaccine hesitancy, a key challenge to achieving widespread vaccination both in Australia and internationally [7, 8]. A 2021 study in Australia found rates of COVID-19-related vaccine hesitancy and resistance increased from 12.7% to 21.7% from August 2020 to January 2021 [9]. Concerns about the COVID vaccine safety and effectiveness, the speed of production and a lack of trust in government are thought to be contributing to this trend internationally [10].

Research suggests that recently-arrived CALD populations (arriving in the last five years), are particularly vulnerable to misinformation and are less responsive to COVID-19 health messaging [11, 12]. While a lack of established support networks, poorer health literacy and language barriers are thought to contribute to this, there is a dearth of research examining this issue in Australia. Further, there is little research examining what methods might be used to overcome vaccination hesitancy in these communities.

Therefore, the aim of this study was to explore the barriers and enablers to COVID-19 vaccination among recently-arrived CALD communities in Melbourne’s outer north and identify strategies to reduce hesitancy in this population.

In 2020, the largest outbreak of COVID-19 in Australia was in Melbourne, accounting for 75% of Australian cases (n = 20,330 on 24^th^ October 2020), and 90% of all deaths (n = 817). Northern Health (NH) is the major provider of public health care in the northern region of Melbourne, Australia and facilitates a mass vaccination hub. Residents living in the region are culturally and linguistically diverse, originating in over 180 countries and speaking over 106 languages [13]. NH’s catchment contains the largest population of refugees who have settled in Victoria in the past five years [14]. While NH’s catchment accounts for 10% of Victoria’s population, one third of Victoria’s COVID-19 cases in 2020 resided in the catchment.

Pfizer (Comirnaty) and AstraZeneca (Vaxzevria) vaccines were provisionally approved for use in Australia in January and February 2021 respectively. Victoria’s vaccination rollout began on February 22 2021, just prior to media reports of a potential safety concern associated with the AstraZeneca vaccine and suspension of its use in several European countries. Interviews for this study were carried out between April and May, 2021. During this time the Australian Technical Advisory Group on Immunisation (ATAGI) updated their recommendations. They advised that Pfizer (Comirnaty) is the preferred vaccine for adults aged under 50 years due to the higher risk of a rare, but potentially life-threatening blood clotting condition associated with the AstraZeneca vaccine [15].

## Methods

This is a qualitative descriptive study based on naturalistic inquiry [16, 17]. We used this methodology as we sought to provide an accurate description of the perceptions of participants. We have reported the methods and results of this study according to the Consolidated Criteria for Reporting Qualitative Research (COREQ) [18].

### Data collection

We sought participation from a sample of individuals working in community organisations that support recently-arrived CALD communities in Melbourne’s north. Purposeful sampling was used to identify both peer workers and health workers within these organisations who worked with recently-arrived communities. Snowball sampling was used to engage additional participants from other organisations following recommendations from those already engaged. In total 16 organisations were invited to partake via email. They included local councils, community health services, settlement services, educational institutions delivering Adult Migrant English Programs (AMEPs), women’s health groups and ethno-specific not-for-profit organisations. Participants were not provided with an incentive to participate. Fifteen participants (73% female) from nine of these organisations agreed to participate (five from health services/networks, five from local governments, three from settlement services, one from an educational institution and one community leader). Participants included two health care workers (refugee nurses), six program/project managers (three of whom also worked as policy advisors for government), one adult migrant education manager, one CEO, four bicultural peer workers and one community leader who work with local CALD communities. Most participants spoke as both representatives of their organisations and of their own experience, however one participant, who is also an active community leader, requested their views to be considered their own.

Nine semi-structured interviews with 15 participants were conducted between 12 April 2021 and 04 May 2021 (one to four participants per interview, median one per interview). Interviews were held via videoconference and ranged from 15 to 45 minutes duration. Interviews were conducted by an experienced researcher initially (RJ) who trained the lead author to carry out subsequent interviews independently. The interviews were transcribed verbatim, de-identified and returned to participants to check for accuracy.

### Interview guide

A rapid literature review was conducted to inform the development of an interview guide. Two electronic databases (PubMed and Web of Science) and grey literature were searched to identify strategies that have been used in Australia to enhance communication with CALD communities throughout the pandemic. This process identified four themes that acted as focus areas for the interviews; access to information, trusted sources, exploring vaccine hesitancy and overcoming vaccine hesitancy.

### Ethics approval

This study received approval from the Northern Health Office of Ethics and Research Governance as a quality improvement project (no. 24.2021).

### Data analysis

Thematic analysis, a reflective and iterative method of qualitative data analysis, was used to identify and group patterns within interview transcripts. A realist epistemology was used that assumed that language accurately reflects and enables participants to articulate their perspectives and experiences [19].

This study utilised Braun and Clarke’s six-step approach to thematic analysis [19]. Initial familiarity with the data was achieved by conducting/attending interviews, transcribing and reading transcripts multiple times. An inductive or ‘bottom-up approach’ was used code the data. To ensure that the interview data was accurately reflected in the conclusions, two authors (KD and RLJ) coded data separately using NVivo. Cross-checks were conducted between coders to ensure accuracy of interpretations and conclusions were verified by a subject specialist (CA). An iterative process was used to identify, review and define themes.

## Results

Four major themes emerged from the analysis; (1) trusted sources, (2) accurate and culturally sensitive information, (3) supported pathways and (4) enablers to vaccination. Each theme and its associated codes are provided in Table 1.

**Table 1:** Themes and Codes

Major Theme	Initial code	No.*
Trusted sources	Authoritative sources	9
	Overseas sources	3
	Peer to peer and communal knowledge sharing	7
	Horizontal dissemination	4
	Vertical dissemination	8
	Trust in authority	2
Accurate and culturally sensitive information	Concerns about vaccine content	2
	Conspiracy theories	7
	Issues with risk communication/scientific translation	4
	Information accessibility	6
	Misinformation	3
	Risk to benefit concerns	5
	Language translation and cultural responsiveness	9
	Safety and efficacy	6
	Dissemination methods	9
	Influencing social norms	3
	Community education forums	6
Supported Pathways	Issues with vaccine access	4
	Social vulnerabilities	4
	One-on-one education	2
	Relationship building	3
Enablers to vaccination	Collective responsibility	4
	Established channels of communication	5
	Established expectations	4
	Gratitude	1
	Previous experiences in pandemics	1
	Highlighting incentives	3

^*^Number of interviews that included initial code

In describing the findings of the interviews, we will use the terms horizontal and vertical information flow to describe the way information is shared within communities. Vertical transmission of information is the delivery of health information to end users from authoritative sources such as professionals or formal organisations and institutions. This differs from horizontal information flow, which refers to sharing of information between peers and within cultural groups.

### Theme 1: Trusted sources

Participants described the importance of identifying and utilising trusted sources to deliver health information to recently-arrived CALD populations in order to reduce vaccine hesitancy. They discussed common places where this population access COVID-19-related health information and the levels of trust associated with them.

*‘Whoever is delivering the messaging and whoever is disseminating the information, it has to be from a trusted source. That’s the number one thing.’ Interview 7, respondent 1*

Participants identified that recently-arrived CALD populations accessed their information from a number of places, including vertical sources, local and overseas media and horizontal knowledge sharing platforms.

#### Barriers to COVID-19 vaccination

While a number of sources for vertical transmission of information were identified as important, a potential barrier identified by participants in two interviews was a lack of trust in authority. Participants attributed this to previous negative experiences with governments and institutions in countries of origin.

*‘They don’t trust the government in general.’ Interview 5, respondent 2*

Horizontal dissemination of information was identified by participants as the primary source of COVID-19 vaccine information for newly-arrived CALD populations. Participants identified that even when this occurs via word of mouth, information often originates from social media. Common social media platforms that participants reported were used within the communities they worked with included Facebook, Viber, WhatsApp and WeChat. These differed slightly depending on cultural group. While younger people were identified as more likely to use social media, the messaging often impacts older people indirectly due to the prevalence of intergenerational living and children assisting parents to make vaccine and health related decisions. Participants reported that this form of information sharing is very important among recently-arrived CALD groups and it is often believed and shared without being critically appraised. As friends and family are often considered a trusted source of information, participants raised the concern that it is hard to bring a counter position to misinformation spread in this manner which may promote hesitancy.

*‘You’re getting people that are very entrenched in their own community and hearing and trusting that community voice above and beyond any other voice that they’re hearing.’ Interview 2, respondent 1*

Bicultural workers are also considered a trusted source of information, however concerns were raised regarding fatigue and overburdening of workers with tasks beyond their professional scope.

*‘When they’ve got one foot in community and one foot in the kind of, professional role, it requires a very delicate balance to be able to support them to do that.’ Interview 7, respondent 1*

#### Proposed strategies to overcome vaccine hesitancy

When asked to identify strategies to reduce hesitancy in recently-arrived CALD populations, participants perceived that utilising a combination of trusted authoritative and peer sources to dissemination COVID-19 vaccine information would be effective. In eight interviews, vertical or top-down information dissemination strategies were recommended. These include engaging health care workers, teachers in AMEPs, religious leaders, community health services, bicultural workers, government agencies, media and cultural support groups to disseminate COVID-19 vaccine information to newly-arrived CALD groups.

*‘But when they’re new arrivals it’s really through the settlement services that they’re attached to… that’s where they’re tapping in to you know, information.’ Interview 2, respondent 1*

Capitalising on preferences for horizontal information dissemination, strategies such as training ‘community ambassadors’ and host families to improve their knowledge about the COVID-19 vaccine and for them to share this information within their community was suggested.

*‘Having a group of volunteers from the community… that actually have done some sort of health literacy training…so you’re basically using that traditional peer-led model of reform.’ Interview 8, respondent 1*

### Theme 2: Accurate and culturally sensitive information

#### Barriers to COVID-19 vaccination

Participants reported that both a lack of accurate, culturally sensitive health information and an abundance of misinformation were key contributors to vaccine hesitancy among recently-arrived CALD populations.

*‘If you, one; don’t have a medical background, two; you don’t speak English, I mean, really, you’re just relying on what Karl Stefanovik and that are pumping out over the TV. Which even that, I listen to that some mornings and just go ‘oh wow, that’s news to me, I didn’t know that’ and it’s not even true some of it.’ Interview 3, respondent 3*

Inadequate risk-communication strategies from authoritative sources were cited as a cause for confusion around safety, with many in the community uncertain about how the vaccine works and the differences between vaccines.

*‘Something that we’ve been hearing from health professionals and other service providers is that there isn’t consistent information going towards them about how to articulate the vaccine and other COVID-related messages to their clients and patients.’ Interview 1, respondent 2*

Anxieties around the speed with which the vaccine was produced, the risk of harmful short- and long-term side effects, particularly for women of reproductive age and children, and a lack of confidence that the vaccines have undergone sufficient testing were considered barriers to vaccination.

*‘The speed of the vaccine is concerning. And unfortunately, now, amplified with the current situation around AstraZeneca.’ Interview 1, respondent 1*

Identified as being of particular importance to this population were concerns about the content of the vaccines, including anxieties about whether the vaccines are halal and whether they were developed using foetal cells.

*‘Another question which is coming up is the stem cell, foetus, cell-medium, aborted foetus question, you know...what is the vaccine made of?’ Interview 1, respondent 1*

Participants described a perceived high-risk to low-benefit ratio as a factor contributing to vaccine hesitancy among CALD populations. Young, healthy individuals, may perceive there is no incentive to receive the vaccine given the negligible community transmission in the region, that they are unlikely to become seriously ill from COVID-19, and there are potentially harmful effects related to the vaccine. In addition, notions that the vaccine does not prevent infection or transmission to others (including loved ones) may reduce any incentives around communal responsibility.

*‘I think because it doesn’t stop people from spreading it, then they’re more likely to be like ‘well I still will have to wear a mask and I’ll still have to do all these things and I…still can’t travel, still have to do quarantine, still have to do this and that.’ Interview 1, respondent 2*

### Proposed strategies to overcome vaccine hesitancy

Strategies to enhance the accuracy and cultural responsiveness of health messages were recommended in all nine interviews to reduce hesitancy. These included using human translators and pretesting health messages with different communities to ensure both accuracy of interpretation and cultural appropriateness.

*‘You can’t really adopt a one-size-fits-all approach to all of those communities and I think it’s about potentially doing some consultation with different communities and figuring out what’s going to work for each of those communities in creating more tailored approaches.’ Interview 1, respondent 2*

Participants recommended utilising a range of information dissemination strategies to reduce vaccine hesitancy among recently-arrived CALD populations. These included running community forums and directly engaging health care workers, teachers in AMEPs, religious leaders, community health services, bicultural workers, government agencies, media and cultural support groups. Providing opportunities for people to share personal experiences of being vaccinated and having community leaders attend cultural events where people can discuss and socialise the issue of vaccination was also suggested by participants.

*‘When you can give them the information that responds to the specific concerns that they may have… they’re more likely to follow through and get vaccinated.’*

*Interview 9, respondent 1*

Participants recommended using a multipronged approach of print and digital strategies to disseminate health information. These include posters in places where recently-arrived CALD community members frequent such as health care waiting rooms, bus stops, restaurants, and in language schools as well as providing information on websites and social media platforms including Facebook, WhatsApp and Instagram. However, websites that do not have clearly accessible links to information in a variety of languages were identified as a barrier to information access for recently-arrived CALD groups.

*‘The reflections around COVID testing and COVID messaging more generally…what has really stood out to us is that they’re using both a mix of print and digital alongside word of mouth.’ Interview 9, respondent 1*

### Theme 3: Supported pathways

#### Barriers to COVID-19 vaccination

Participants highlighted a number of challenges faced by members of recently-arrived CALD communities in regards to vaccine information and uptake which require nuanced and supportive service delivery approaches. Social vulnerabilities common to recently-arrived CALD groups such as language barriers, technological barriers, social isolation, vulnerable living arrangements and a history of trauma were raised as barriers to accessing and understanding information in a timely manner.

*‘I think we take for granted the barriers that are put up…We don’t see the barriers that are there for people who don’t speak English, [who] have maybe a temporary visa or have had a traumatic past or intergenerational trauma.’ Interview 7, respondent 1*

Logistical and access issues were identified as a barrier to vaccine uptake for those members of the CALD community willing to be vaccinated. These include uncertainty about eligibility and where to access vaccines, as well as uncertainty from providers about supply.

*‘We have these calls every fortnight about service providers and the network and…members are going ‘where do you get it? I don’t know how to get it.’’*

*Interview 1, respondent 1*

#### Proposed strategies to overcome vaccine hesitancy

Participants perceived that providing opportunities for individualised clinical advice would help to overcome barriers to vaccination. Delivering and promoting a service where one-on-one education can take place at the point of vaccine delivery was suggested as a strategy to enable people to explore individual concerns.

*‘A lot of the asylum seeker health services that we work with have been saying that the education and the service delivery go hand in hand.’ Interview 1, respondent 2*

Improving the cultural responsiveness of Northern Health’s vaccine service delivery through active service promotion and supported engagement pathways was recommended, particularly for refugee and asylum seeker populations.

*‘They actually need a supported re-engagement pathway or, where they don’t come to an appointment, that there’s an active phone call that’s made to them.’*

*Interview 9, respondent 1*

Participants felt that a commitment to building and strengthening relationships with local organisations who work with newly-arrived refugees and asylum seekers was needed to enhance long-term, culturally sensitive service provision to members of local CALD communities.

*‘We need to be doing a lot more…and not in times of crisis, in times of…normal circumstance, we need to be building those relationship [sic] and building that trust. So that when we do have situations like this… we are considered a trusted source.’ Interview 7, respondent 1*

### Theme 4: Enablers to vaccination

Capitalising on community beliefs and experiences to facilitate vaccination among recently-arrived CALD populations was identified by participants as an important strategy for engagement. These included recognising recently arrived communities have a strong sense of collective responsibility, high acceptance of immunisation as a requirement of entry into Australia, previous experience in pandemics and a general sense of gratitude to be living in Australia where COVID case numbers are low relative to many other countries.

*‘Of course they would like to protect themselves and protect their families, community and people around them and the rest of the community.’ Interview 6, respondent 1*

Participants identified existing networks can be leveraged to improve vertical information dissemination. These included collaborating with a range of government agencies and health services as well as local networks through AMEPs, support groups, church groups and the social media networks of bicultural workers.

*‘It’s about really leveraging those networks and connection that is already in place.’ Interview 4, respondent 1*

A number of incentives that are important to recently-arrived CALD populations were described with participants recommending they be highlighted in messaging to increase vaccine uptake. These include benefits of individual vaccination to the broader community, an increased likelihood of family reunification and reduced economic impacts of lockdowns.

*‘Economically, they’re going to want to know that COVID won’t hit them again hard.’ Interview 2, respondent 1*

## Discussion

This study provides important learnings from the experiences of participants from community organisations working with recently-arrived CALD communities in the multicultural outer north of Melbourne.

This study identified the importance of identifying and utilising trusted sources in the delivery of health information to recently-arrived CALD populations. We found that recently-arrived CALD groups are accessing information from a range of horizontal (peer-to-peer) and vertical (authoritative) sources. A strong theme that emerged from the data is a paradox between trust and credibility in relation to these two forms of information gathering. The data revealed that recently-arrived CALD communities place greater trust in information they gain through horizontal, less credible sources, including family, friends and people from their community both locally and overseas. This aligns with a recent systematic review [20] that found that social norms and attitudes of peers strongly influence vaccine uptake among migrant and refugee communities.

We found that often when word of mouth is a primary source of information, the information originates from social media platforms. This is consistent with findings from a recent New South Wales Council of Social Services (NCOSS) study [21] that found that ‘Facebook’ and ‘other forms of social media’ were the two largest sources of COVID-19 vaccine information among CALD communities in NSW. Information on social media may lack credibility, and in fact may be deliberately misleading, facilitating the spread of misinformation [22].

Conversely, while information shared vertically from authoritative sources such as government, media and medical institutions may be more credible, a lack of cultural responsiveness in health message development coupled with some distrust in authority means that it is less likely to be an effective communication method for recently-arrived CALD groups. This is supported by NCOSS’s study [21] which indicated that formal channels such as government websites are not the preferred source of information for CALD communities in NSW as they can be difficult to navigate and are not tailored to people with low levels of health literacy. Our study highlights the need for increased efforts from governments and health services to build sustainable relationships and collaborate with CALD communities to ensure that credible sources also become trusted ones.

Language barriers and difficulty navigating health care systems have been identified as barriers to vaccine uptake among refugee and migrant communities globally [23]. Additionally, it has been established that identifying trusted sources of information is an important element of effective communication with CALD populations [12, 24, 25]. While faith leaders, settlement workers and community leaders are commonly recognised as trusted sources, for the first time this study introduces the importance of teachers in language acquisition programs. Teachers in these programs are often in positions of trust, and so are well placed to support recently-arrived CALD communities to overcome barriers to vaccine acceptance.

Bicultural workers have been utilised to improve public health communication throughout the pandemic which has raised a number of ethical concerns regarding overburdening, over-reliance and subsequent burnout [12, 24]. Bicultural workers use both their proficient language skills and their shared cultural skills, knowledge and values to act as a bridge to negotiate and communicate between communities and health or government agencies [26]. Findings from our study indicate that these workforce roles are trusted within communities and growth in this workforce to reduce burden on the small numbers currently employed should be considered to assist with future public health efforts in Australia.

Technology and social media have facilitated the rapid spread of large volumes of mis- and disinformation, contributing to the COVID-19 infodemic [27]. A recent Australian study found the following factors to be associated with belief in misinformation; younger age, male gender, lower education level, language other than English spoken at home, low perceived threat of COVID-19 and distrust in government [22]. This may contribute to vaccine hesitancy among CALD populations who often experience lower levels of health literacy and may struggle to critically evaluate the overabundance of health information they receive [28]. Our study supports the premise that excessive information and misinformation and lack of clear, accurate and easily accessible information is contributing to vaccine hesitancy. It also highlights that a shortage of risk- and science-communication skills in health workers and bicultural workers may exacerbate this issue. Strategies suggested to overcome this include employing a multipronged communication approach including print, digital and outreach methods, and training key personnel to improve risk communication skills. Other recommendations align with those from international literature and include considering health literacy principles and cultural responsiveness in the development of tailored health messages and services for CALD communities [23].

A recent study looking at COVID-19 vaccine hesitancy suggests that many people who are vaccine hesitant do not hold strong anti-vaccination views and may be amenable to vaccination [10]. Our study suggests this may apply to recently-arrived CALD communities with participants indicating that only a minority hold anti-vaccination sentiments. Additionally, it was suggested that most of those labelled vaccine hesitant are quite receptive to the concept of vaccination, however have specific questions or concerns they need addressed in order to accept the COVID-19 vaccine. This is supported by NCOSS’s study [21] that suggests that vaccine hesitancy among CALD populations in NSW may better described as low motivation with participants choosing to take a ‘wait-and-see’ approach. Our study indicates that facilitating opportunities to contextualise information through community forums, where people can ask questions and share personal stories, and allowing for one-on-one education prior to vaccine delivery may address these issues.

A strength of this study is that it was conducted in Australia and therefore provides new perspectives relevant to the Australian context. There has been a general decline in COVID-19 vaccination rates since mandates have been lifted. The rates for third doses in people aged 16 and over are 72.2% compared to 97.2% and 95.8% for first and second doses respectively (as at November 2, 2022) [29]. Given the risk of future variants of the virus and the possibility that further vaccination will be advised, keeping the community vigilant is an ongoing challenge. Our study identifies a range of methods that may be utilised to continue to engage people and will be important to protect those most vulnerable to deterioration.

The most significant limitation of this study is that views about vaccination seeking behaviour of recently-arrived CALD populations were collected indirectly from representatives of organisations and community leaders working with these communities. Therefore, it is important to note that participants views are their own interpretations of what they have heard or witnessed, rather than direct experiences of the target population. Saturation may not have been reached in some themes and ideas to reduce vaccine hesitancy in CALD populations may exist that were not captured in the data set. Researcher bias is an inherent limitation of thematic analysis as the researcher’s worldview impacts their interpretation of the data. It is important to note that both authors who conducted the thematic analysis (KD and RLJ) are not from CALD backgrounds, therefore did not have a lived experience to draw upon when interpreting the findings. The use of purposive and snowball sampling may have limited the range of views from participants.

## Conclusion

This study found that participants perceived a lack of accurate, culturally sensitive health information and service provision as key barriers to vaccination in recently-arrived CALD communities in Melbourne’s outer north. Participants identified a range of enablers to vaccination in this population including established channels of communication, a strong sense of collective responsibility and general acceptance of vaccination. Specific strategies to reduce vaccine hesitancy in recently-arrived CALD communities were identified such as identifying and utilising trusted sources to disseminate information, tailoring health messages, providing opportunities to contextualise information and modifying service delivery to enhance cultural sensitivity. The paradox between trust and credibility of sources used by CALD populations to gather COVID-19 vaccine information highlights the need for increased efforts from health and government agencies to build sustainable, collaborative relationships with CALD communities. Further consultation with specific groups is required to develop culturally appropriate, tailored approaches to public health communication to reduce vaccine hesitancy.

## Data Availability

The data that support the findings of this study are available from the corresponding author upon reasonable request.

## Abbreviations

AMEP: Adult Migrant English Programs
CALD: Culturally and Linguistically Diverse
NCOSS: New South Wales Council of Social Services
